# Preventing Alcohol-Related Problems on College Campuses: Summary of the Final Report of the NIAAA Task Force on College Drinking

**Published:** 2004

**Authors:** Robert F. Saltz

**Affiliations:** Robert F. Saltz, Ph.D., is a senior research scientist at the Prevention Research Center, Pacific Institute for Research and Evaluation, Berkeley, California

College administrators and their prevention staff face numerous challenges when attempting to reduce the prevalence and severity of alcohol consumption and alcohol-related harm on their campuses. For example, drinking, and particularly binge drinking have been shown to be pervasive and persistent behaviors among college students (Wechsler et al. 2000). In addition, until a few years ago research assessing the effectiveness of various prevention approaches in the college setting was scarce, making it difficult to identify effective measures. In recent years, however, several studies have looked more closely at approaches to preventing college drinking. For example, Dowdall and Wechsler (2002), Borsari and Carey (2001), Perkins (2002*a*), and Berkowitz (2004) reviewed or analyzed prevention approaches among college populations. Another important contribution was the final report from the National Institute on Alcohol Abuse and Alcoholism (NIAAA) Task Force on College Drinking (NIAAA 2002), which reviewed epidemiological and intervention research on college drinking and issued recommendations for prevention strategies. This article describes the motivation for focusing on college student drinking and summarizes the Task Force’s findings and recommendations.

## Why Target College Student Drinking?

Given the all-too-common reports in the press of occasional tragic deaths and of mass celebrations or riots among college students that are accompanied by alcohol consumption, the answer to this question may seem obvious. But these singular events do not accurately reflect the actual prevalence of death and injury associated with alcohol use among college students. Based on epidemiological data from a variety of sources, Hingson and colleagues (2002) generated the following estimates of the consequences of college student drinking:

- More than 1,400 college students die annually in alcohol-related events, primarily traffic crashes.
- More than 2 million college students (of a total of 8 million) occasionally drive under the influence of alcohol, and more than 3 million students ride with a drinking driver.
- More than 500,000 students annually suffer unintentional injury under the influence of alcohol.
- More than 600,000 students annually are hit or assaulted by another student who has been drinking.

In addition to these acute consequences of drinking, evidence suggests that alcohol consumption can lead to longer term cognitive impairment (Spear 2002; White 2003).

The college environment itself (specifically, such factors as peer influence and alcohol availability) may contribute to college students’ risk of alcohol-related harm. O’Malley and Johnston (2002) found that although college-bound high school students drink less than their peers, their alcohol consumption surpasses that of their noncollege peers during the college years, only to decrease again after they finish college. In light of these observations, drinking among college students deserves special attention.

## The NIAAA Task Force on College Drinking

The NIAAA Task Force on College Drinking commissioned several review papers on various aspects of drinking among college students (e.g., drinking patterns and consequences of alcohol consumption).^1^ With respect to prevention research, however, the Task Force found that studies evaluating prevention approaches focused mostly on interventions aimed at individual student drinkers rather than on interventions aimed at entire college populations (i.e., universal interventions).

The Task Force’s findings regarding the efficacy of individual-level interventions have been reviewed and summarized by Larimer and Cronce (2002), who distinguish between educational or awareness programs, cognitive-behavioral interventions, and motivational enhancement techniques. With one or two exceptions, this review found little evidence to support the effectiveness of purely educational or awareness programs. Newer approaches combining provision of information with other components, such as motivational enhancement, await evaluation and may be found to be more successful.

Cognitive-behavioral skills training programs attempt to teach skills relevant to moderating alcohol consumption, including those specific to drinking (e.g., monitoring one’s consumption or gauging one’s blood alcohol levels) and more general life skills, such as stress management. These programs also can include components such as clarification of values, information, and/or education.

One cognitive-behavioral approach focuses on identifying students’ expectancies regarding alcohol’s effects, because studies have shown that a substantial portion of alcohol’s effects is attributable to such expectancies rather than to alcohol’s physiological effects. Research indicates that focusing on expectancies impacts the drinking behavior of students, particularly males.

Larimer and Cronce (2002) concluded that the most promising interventions incorporate several components, such as training in drinking skills and life skills, self-monitoring, and challenges to students’ expectancies.

Brief interventions—which include alcohol information, skills training, and personalized, nonjudgmental feedback to enhance motivation to change—can be effective in both individual and group formats.

## NIAAA Task Force Recommendations

The NIAAA Task Force summarized its findings and recommendations in a comprehensive report (NIAAA 2002). At the time the report was written, the interventions described above represented nearly all that had been rigorously evaluated with college students. However, the Task Force expanded the scope of the recommendations by including universal prevention efforts that had been evaluated in other community settings and could reasonably be extended to college settings as well as those already adopted by some colleges without being formally tested. The recommendations were organized in four “tiers” based on both the interventions’ relevance to college student drinking and the degree to which they are supported by empirical evidence. Although this classification is not universally accepted and may have to be modified in response to more recent research, it can help college administrators and researchers to choose the most promising approaches.

### Tier 1

Based on the findings described in the previous section, the Tier 1 category included strategies that show evidence of effectiveness with college students, including:

- Combinations of cognitive-behavioral skills training with norms clarification and motivational enhancement interventions.
- Brief motivational enhancement interventions.
- Interventions challenging alcohol expectancies.

### Tier 2

This category includes strategies that research shows have been successful with general populations and could be applied to college settings, including efforts either to restrict the availability of alcohol or to create an environment supportive of such restrictions. These universal strategies are critical because alcohol-related harm to society can be attributed not only to the heaviest drinkers but also to the large numbers of light and moderate drinkers (Gruenewald et al. 2003). Tier 2 strategies could include approaches involving minimal legal drinking age (MLDA) laws (e.g., increased enforcement of MLDA laws) and other alcohol-related criminal and administrative measures such as:

- Implementation, increased publicity, and enforcement of laws to prevent alcohol-impaired driving.
- Restrictions on alcohol retail outlet density.
- Increased prices and excise taxes on alcoholic beverages.
- Responsible beverage service policies in social and commercial settings.
- Campus and community coalitions of all major stakeholders to implement these strategies effectively.

The Tier 2 interventions have not been evaluated on college campuses, at least in part because such measures are challenging to implement and studies are difficult to design. Efficacious community-level prevention interventions, such as the Massachusetts Saving Lives program (Hingson et al. 1996), Communities Mobilizing for Change on Alcohol (Wagenaar et al. 2000), and the Community Trials Project (Holder et al. 2000), however, can guide future college-based efforts.

### Tier 3

Tier 3 consists of strategies with logical and theoretical promise that require more comprehensive evaluation. These strategies could prove effective in future studies, and some already are highly regarded by prevention program professionals and college administrators. The Task Force Report suggested the following Tier 3 strategies:

- Marketing campaigns to correct student misperceptions of peer alcohol use, sometimes called “social norms marketing” or normative education (see Perkins 2002*b*). (This strategy already is widely used; evaluation reports will be available in the near future.)
- Consistent enforcement of campus alcohol policies.
- Provision of safe rides for students who drink too much to drive.
- Regulation of happy hour promotions.
- Information for new students and their parents about alcohol use and campus policies.
- Other strategies to address high-risk drinking, such as offering alcohol-free residence halls and social activities or scheduling classes on Fridays to reduce Thursday night parties.

### Tier 4

Tier 4 strategies include those with “evidence of ineffectiveness,” such as simple educational or awareness programs used alone, without any other strategies or components. The Task Force warned against the use of breathalyzers to give students information about their level of impairment, because this approach has produced negative results (i.e., students have used the information as a challenge to reach higher levels of intoxication).

## Future Research and Applications

Although the NIAAA Task Force and others have identified some strategies to prevent college drinking, more research and evaluation are needed to identify more effective approaches for college administrators to add to their repertoire. Additional research needs to focus on how to implement successful universal campus or community interventions. Although researchers have achieved some successes in implementation (at least, judging from the positive examples of Tier 2 strategies), many of these interventions have not been subject to systematic research. Few researchers are developing measures of organizational or community “readiness” based on an underlying theory or hypothesis about what facilitates implementation (see Oetting et al. 1995). Nevertheless, developing general models of organizational or community change that are applicable to alcohol prevention is critically important to the design of reliable strategies that will keep college students safe from harm.

## Footnotes

1 These reviews are available at www.collegedrinkingprevention.gov.
